# The Evolution of Neurofilament Light Chain in Multiple Sclerosis

**DOI:** 10.3389/fnins.2021.642384

**Published:** 2021-04-06

**Authors:** Carolina Ferreira-Atuesta, Saúl Reyes, Gavin Giovanonni, Sharmilee Gnanapavan

**Affiliations:** ^1^Department of Neurology, Icahn School of Medicine at Mount Sinai, New York, NY, United States; ^2^Department of Neurology, Hospital Universitario Fundación Santa Fe de Bogotá, Bogotá, Colombia; ^3^The Blizard Institute, Barts and The London School of Medicine and Dentistry, Queen Mary University of London, London, United Kingdom; ^4^Department of Neurology, The Royal London Hospital, Barts Health NHS Trust, London, United Kingdom

**Keywords:** multiple sclerosis, biomarkers, individualized medicine, prediciton, neurofilament light, demyelination

## Abstract

Multiple sclerosis (MS) is an autoimmune, inflammatory neurodegenerative disease of the central nervous system characterized by demyelination and axonal damage. Diagnosis and prognosis are mainly assessed through clinical examination and neuroimaging. However, more sensitive biomarkers are needed to measure disease activity and guide treatment decisions in MS. Prompt and individualized management can reduce inflammatory activity and delay disease progression. Neurofilament Light chain (NfL), a neuron-specific cytoskeletal protein that is released into the extracellular fluid following axonal injury, has been identified as a biomarker of disease activity in MS. Measurement of NfL levels can capture the extent of neuroaxonal damage, especially in early stages of the disease. A growing body of evidence has shown that NfL in cerebrospinal fluid (CSF) and serum can be used as reliable indicators of prognosis and treatment response. More recently, NfL has been shown to facilitate individualized treatment decisions for individuals with MS. In this review, we discuss the characteristics that make NfL a highly informative biomarker and depict the available technologies used for its measurement. We further discuss the growing role of serum and CSF NfL in MS research and clinical settings. Finally, we address some of the current topics of debate regarding the use of NfL in clinical practice and examine the possible directions that this biomarker may take in the future.

## Introduction

The clinical use of biomarkers in the diagnostic work-up of neurodegenerative diseases has significantly increased in the last decades (Beart et al., 2017). Screening, drug development, early diagnosis, individualized therapy, and accurate prognosis are some of the key factors driving the need of identifying objective and quantifiable markers and developing sensible biomedical tools for their measurement.

Multiple sclerosis (MS) is an autoimmune, inflammatory neurodegenerative disease characterized by demyelination and neurodegeneration of the central nervous system (CNS; Reich et al., 2018). The pathomechanisms vary greatly from person to person, resulting in different pathological phenotypes, clinical presentations, progression trajectories, and treatment responses (Lassmann et al., 2001; Gnanapavan and Giovannoni, 2015; Gafson et al., 2017; Reich et al., 2018; Dobson and Giovannoni, 2019; Ziemssen et al., 2019). Current diagnostic criteria and clinical management depend on radiological markers (i.e., MRI), clinical status (i.e., disease activity and disability), and immunological parameters suggestive of inflammation (i.e., oligoclonal IgG bands; Thompson et al., 2018). However, these markers often fail to predict individual relapse rates, disability progression and therapy response (Håkansson et al., 2017). A meta-analysis showed that the number and volume of T2 lesions on MRI correlated poorly with clinical presentation and disease progression (Li et al., 2006). Similarly, the MAGNIMS study group concluded that clinical and MRI activity in isolation was not enough to evaluate treatment response (Gasperini et al., 2019). There is, therefore, an increased need to identify and validate biomarkers that could be used as surrogate measures for clinical endpoints in a more individualized manner (Giovannoni, 2006; Gnanapavan et al., 2013; Pachner et al., 2019; Gaetani et al., 2019a; Ehrenberg et al., 2020).

One of the hallmark features of MS, and which correlates highly with disability, is axonal damage, and loss (Trapp et al., 1998; de Stefano et al., 2002; Filippi et al., 2003; Pascual et al., 2007). Recent reports have demonstrated that people with MS (pwMS) have up to 60% reduction in axons at all spinal levels involving all fibers regardless of their diameter (Tallantyre et al., 2009; Petrova et al., 2018). This axonal loss has been associated with brain and cervical atrophy (Bjartmar and Trapp, 2001; Lee et al., 2014; Petzold, 2015; Barro et al., 2018), cortical thinning (Popescu et al., 2015), disability (Siffrin et al., 2010; Domingues et al., 2019), fatigue (Tartaglia et al., 2004), cognitive dysfunction (Gadea et al., 2004), and suboptimal response to therapy. Quantifying and monitoring axonal loss could be a reliable marker of MS progression, disability and treatment response (Novakova et al., 2017a, 2018).

## Neurofilaments as Biomarkers in MS

Neurofilaments (Nf) are structural scaffolding proteins of the axonal cytoskeleton. Nf are essential for stability, radial growth and maintenance of axonal caliber and electrical-impulse transmission (Yuan et al., 2012; Gnanapavan and Giovannoni, 2015; Ziemssen et al., 2019). Given that Nf are involved in axonal radial growth, larger myelinated axons express significantly more Nf (Yin et al., 1998). Nf are composed of four subunits: neurofilament heavy, median and light polypeptides [NfH, NfM, and Neurofilament Light chain (NfL), respectively], as well as α-internexin (Int). Each subunit possesses a particular molecular mass (68 kDa for NfL, 150 kDa for NfM, and 190 to 210 kDA for NfH) and their relative concentration is uneven, however, NfL is the most abundant and soluble of the subunits. Under normal conditions and in a non-linear, sex-, and age-dependent manner (Gisslén et al., 2016), Nf are constantly released from axons (Disanto et al., 2017; Bridel et al., 2019), reflecting normal aging (Khalil et al., 2020). However, during axonal damage, Nf are released in larger quantities into the extracellular space, the cerebrospinal fluid (CSF), and eventually into the blood, where concentrations are 40-fold lower than in the CSF (Gaetani et al., 2019a). Overall, measurement of Nf levels indicate the extent of axonal damage, and therefore, is a bulk marker of disease activity (Lycke et al., 1998; Bergman et al., 2016; Disanto et al., 2017; Novakova et al., 2017a; Håkansson et al., 2018; Khalil et al., 2018; Cantó et al., 2019; Domingues et al., 2019; Varhaug et al., 2019; Gaetani et al., 2019b).

Several characteristics make Nf, and particularly NfL, a good biomarker of neurodegeneration. Firstly, NfL can be objectively measured and quantified, it is highly sensitive to neurodegenerative processes and its concentration changes as the disease worsens or improves (Disanto et al., 2017). Numerous studies have shown that NfL levels increase during MS relapses and correlate with MRI lesion development (Disanto et al., 2016, 2017; Novakova et al., 2017b; Barro et al., 2018), disease activity, disability and disease progression (Thebault et al., 2020). Secondly, NfL measurement is safe for the patient and NfL levels are relatively easy to detect. Emerging technologies allow for rapid, simple and minimally invasive quantification methods. This allows periodic measurements, and easier sampling acquisition and storage. Last but not least, several clinical trials have included NfL as an outcome measure and have shown that disease modifying therapies (DMTs) significantly reduce NfL levels compared with placebo (Gunnarsson et al., 2011; Axelsson et al., 2014; Christensen et al., 2014; Kuhle et al., 2015; Romme Christensen et al., 2019). This finding makes NfL a valuable outcome measure in clinical trials (Axelsson et al., 2014).

Yet, there are some important caveats that should be considered when quantifying and using NfL measurements in clinical practice. Importantly, NfL is not specific for MS. Neurodegenerative diseases such as prion diseases, amyotrophic lateral sclerosis (ALS), Alzheimer’s disease, Parkinson’s disease, Hungtington’s disease, and traumatic brain injury have all demonstrated increased levels of NfL (Bridel et al., 2019; Gaetani et al., 2019a). Other studies have shown that different subunits might reflect different neurodegenerative processes (Zucchi et al., 2020). For example, NfH, which undergoes extensive phosphorylation and influences the transportation dynamics along axons, is particularly specific for ALS (Xu et al., 2016). Additionally, NfL levels do not provide information on the specific location of axonal damage (Disanto et al., 2017), and a growing body of evidence has shown that NfL is also elevated in individuals with peripheral nerve disease (Sandelius et al., 2018; Hyun et al., 2020), which further limits its use as a diagnostic biomarker. Moreover, standardized normal cut-off values are still lacking, and despite longitudinal measurements being preferred for clinical decision-making, optimal sampling frequency and thresholds are yet to be defined (Domingues et al., 2019; Bittner et al., 2020). It has been documented that NfL levels depend on age, sex and, presumably, body mass index and blood volume (Manouchehrinia et al., 2020a). But as yet, no normative values accounting for confounders have been established.

In the case of individuals with established MS, some authors have reported elevated NfL as the sole indicator of disease activity in people with progressive MS (PMS) vs relapsing remitting MS (RRMS) (Reyes et al., 2020), while others have found greater levels of NfL in RRMS vs PMS (Martin et al., 2019), and that NfL levels at time of diagnosis correlated with long- term progression from RRMS to PMS (Bhan et al., 2018). A recent systematic review concluded that associations with current or future disability are inconsistent, and that there is no evidence of NfL being a responsive marker of purportedly neuroprotective treatments (Williams et al., 2020). More and longer studies are needed examining the evolution and significance of NfL as the disease progresses.

## Correlation of NfL Levels With Radiological and Clinical Findings

The correlation between NfL levels and clinical and radiological findings has been widely reported in the literature. Clinically, it has been shown that NfL levels peak at 3–4 weeks after a clinical relapse and remain elevated for the next 6–12 months (Novakova et al., 2017b; Damasceno et al., 2019). It has also been shown that the cumulative number of relapses in the past 12 months are the main predictor of high NfL levels (Bergman et al., 2016; Kuhle et al., 2019a). In addition, high levels of NfL correlate with brain atrophy and spinal cord volume loss, even in the absence of MRI activity (Arrambide et al., 2015; Kuhle et al.,2016b,a; Disanto et al., 2017; Novakova et al., 2017b; Barro et al., 2018; Chitnis et al., 2018; Piehl et al., 2018; Siller et al., 2019; Khalil et al., 2020).

The predictive value of NfL levels as an independent predictor of conversion from clinical isolated syndrome (CIS) or radiological isolated syndrome (RIS) to MS has been studied. Hakason and colleagues compared the predictive role of NfL and other molecules for MS conversion in individuals presenting with CIS and found that CSF NfL (cNfL) at baseline was the best predictive biomarker (Håkansson et al., 2017). Gaetani et al. (2019b) concluded that a cNFL cut off value of 500 pg/mL was able to predict conversion to MS in individuals presenting with isolated clinical events. Importantly, cNfL levels have been found to be an independent predictor of conversion from RIS to MS (hazard ratio = 1.03, *P* = 0.003; Matute-Blanch et al., 2018), as well as when combined with oligoclonal bands (OCBs; Fyfe, 2018). Moreover, a recent nested case-control examining NfL levels in blood (bNfL) samples from asymptomatic United States military personnel that later developed MS found an association between baseline or presymptomatic levels and long-term risk of developing MS (*p* = 0.008; Bjornevik et al., 2020). Even though similar results have been found elsewhere (Martínez et al., 2015; van der Vuurst de Vries et al., 2019; Dalla Costa et al., 2019a), some studies have reported only a weak predictive value (Arrambide et al., 2016; Disanto et al., 2016).

Axonal damage resulting in brain volume loss plays a key role in long term disability (Furby et al., 2008; Domingues et al., 2019; Marciniewicz et al., 2019). As expected, bNfL levels have been used as potential predictors of long-term progression according to the Expanded Disability Status Scale (EDSS; Häring et al., 2020), however, findings are inconsistent. A study that included 607 individuals with MS followed up for 12 years, showed a significant increase of 80% in bNfL levels per increase in EDSS score (Cantó et al., 2019). Yet, they did not observe an association with long term disability progression. Disanto and colleagues reported increased worsening of EDSS and increased relapse rates at 2 years in individuals with CIS and RMS with bNfL levels above the 80th percentile compared to healthy controls (Disanto et al., 2017). Later on, they reproduced their findings adjusting for other predictors such as T2 lesion load and observed a modest association (OR 2.79) for bNfL above the 90th percentile (Barro et al., 2018). Likewise, Anderson et al. (2020) found a non-significant association between bNfL and EDSS at 5 years in a cohort of 164 pwMS. Moreover, Chitnis et al. (2018) did not find any association between 10-year EDSS scores and bNfL levels collected within 5 years of disease onset. Interestingly, they reported that bNfL correlated with 10-year MRI markers including T2-weighted lesion volume and atrophy. A composite model including bNfL and T2 lesion load was, therefore, deemed robust for predicting long term disability (Chitnis et al., 2018). A similar conclusion was reached by Häring et al. (2020) and Bittner et al. (2020), who measured bNfL and MRI lesion load in 814 individuals with CIS or newly diagnosed MS from 22 centers across Germany.

The correlation between disability and cNfL has also been studied. A randomized controlled trial extension study including 235 pwMS reported that cNfL levels measured at 2 years and bNfL levels measured at 3 years were associated with EDSS scores at 8 years (Kuhle et al., 2019b). However, these levels were measured after beginning of treatment with intramuscular interferon β-1a. Similar results were found elsewhere (Manouchehrinia et al., 2020b). Similar to findings on bNfL, a group reported a correlation between cNfH levels and brain and spinal cord atrophy after 15 years of follow up, but not with EDSS (Petzold et al., 2016). A possible explanation for the modest association between Nf and disability progression is the fact that most studies have not controlled for these potential confounders, such as treatment with DMTs (Anderson et al., 2020).

The association of NfL and non-motor symptoms of MS (e.g., cognition, psychological disorders, and fatigue) has been examined. While some studies reported a significant inverse association between NFL levels and cognitive function (Tortorella et al., 2015; Gaetani et al., 2019c) and long-term fatigue (Chitnis et al., 2018), others did not find any association (Håkansson et al., 2019; Aktas et al., 2020). Cognitive symptoms and fatigue in MS are strongly associated with sleep quality, depression, DMT, disease duration and severity, and lesion localization (Rocca et al., 2014; Berard et al., 2019; Palotai et al., 2019). Therefore, future studies examining the association between NfL and non-motor symptoms should control for these confounders.

## NfL and MS Mimics

Neuromyelitis optica spectrum disorder (NMOSD) and myelin oligodendrocyte glycoprotein antibodies (MOG-Ab) associated disorders (MOGAD), have clinical features that overlap with MS which makes misdiagnosis possible (Alkhasova et al., 2020). Around one third of individuals with NMSDO and MOGAD are seronegative for the highly specific pathognomonic antibodies: aquaporin-4 (AQP-4) and MOG-Ab, which further increases the potential for misdiagnosis and wrong treatments (Fujihara, 2019). Recent studies have analyzed NfL levels in these individuals to see whether threshold values could help differentiate MS from other idiopathic inflammatory demyelinating disorders. A study from China found no significant differences between bNfL levels in pwMS vs CIS vs NMOSD (Peng et al., 2019). In addition, and in line with the underlying pathophysiology of NMOSD vs MS (astrocytic vs axonal involvement, respectively), they found no correlation between bNFL levels and serum AQP-4 (Peng et al., 2019). Importantly, some have reported that high bNfL levels in seropositive NMOSD and MOGAD are associated with a more malignant course of the disease (*p* < 0.05), presumably reflecting concomitant axonal damage (Mariotto et al., 2017). This association between bNfL levels and disability in NMOSD (Watanabe et al., 2019; Kim et al., 2020) and MOGAD (Mariotto et al., 2019) has been found elsewhere. Recently, Watanabe et al. (2019) reported that higher sGFAP/bNfL ratio at relapse was sensitive (73%) and specific (75.8%) to differentiated NMOSD from MS. However, more studies are needed for this to be translated to the clinical setting. Moreover, Lee and colleagues found that NFL levels in NMOSD vs MS vary more significantly according to age (Lee et al., 2020), which emphasizes the need for age-controlled normative values.

## Biomarker Technology

The first assay to measure NfL was developed by Rosengren et al. (1996). This was an ELISA assay based on polyclonal antisera, which was later upgraded to a highly specific assay based on monoclonal antibodies (47:3 and 2:1) against NfL epitopes (Norgren et al., 2002). More recently, Gaetani et al. (2018) generated two novel monoclonal antibodies (NfL21 and NfL23) and a new ELISA assay, which has expanded the currently available methods to measure NfL. NfL ELISA, which is commercially available as NF-light^®^ ELISA kit; UmanDiagnostics AB, Umeå, Sweden, allows for a fast quantification of cNfL with a low sample volume (50 μL). Additionally, it shows good stability after handling and storage (Norgren et al., 2002). The main disadvantage is its low sensitivity for quantifying bNFL. In comparison to CSF sampling, blood sampling is less invasive. Methods such as electrochemiluminescence (ECL)-based immunoassays, which use the luminescence produced during electrochemical reactions of specific antibody combinations, are more sensitive ways of measuring NfL in blood (Li and Mielke, 2019). They are also affordable and require smaller sample volumes (Limberg et al., 2015; Kuhle et al., 2016a). Nevertheless, it has been shown that ECL-based methods are not sufficiently sensitive to detect the lowest concentrations in MS (Hendricks et al., 2019; Li and Mielke, 2019), which limits its utility.

Quantification of bNfL and cNfL has become optimized with the development of the ultrasensitive Simoa^®^ (Quanterix; Hendricks et al., 2019; Gaetani et al., 2019a). Simoa is 125- and 25-fold more sensitive than conventional ELISA and ECL-based assays, respectively. Notably, it can detect a concentration as low as 0.1 pg/mL of protein (Kuhle et al., 2016a). Kuhle et al. (2016a) quantified and compared bNfL levels across the mentioned technologies and found that 55% of ELISA serum measurements and 60% of ECL measurements were below sensitivity when compared to Simoa. A number of studies have shown that Simoa bNFL has good correlations with clinical and radiological findings (Disanto et al., 2017; Novakova et al., 2017b; Piehl et al., 2018), which supports its potential role as a surrogate biomarker in MS. Additionally, Simoa can also detect tau and other proteins associated with neurodegeneration to similar sensitivities, which widens its utility (de Wolf et al., 2020).

**FIGURE 1:**
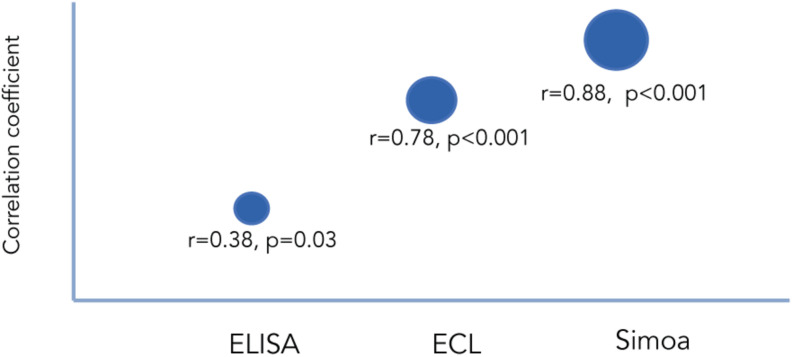
Correlation between cNfL and bNfL across different technologies as reported by Kuhle et al. (2016a). Correlation coefficients between cNfl and bNfL levels measured with ELISA, ECL, and Simoa.

**FIGURE 2:**
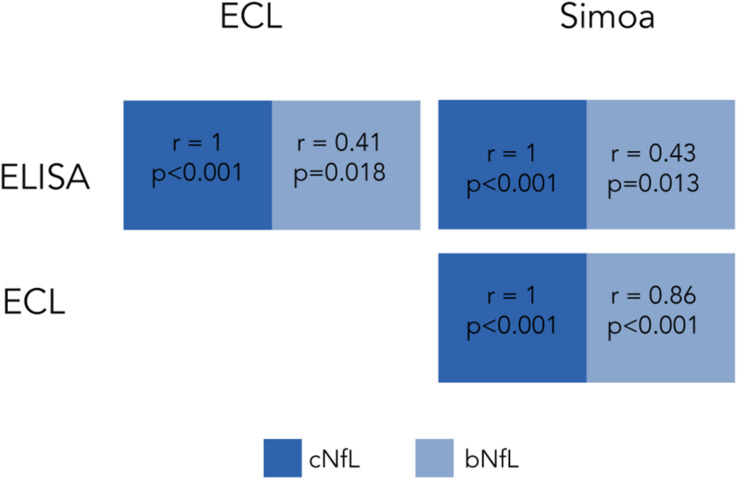
Correlation of cNfL and bNfL across different technologies as reported by Kuhle et al. (2016a). Correlation coefficients of cNfl and bNfL levels across ELISA, ECL and Simoa.

Several studies have examined the correlation between cNfL and bNfL levels between the different technologies. Kuhle et al. (2016a) compared the three mentioned technologies using matched CSF and blood samples from individuals with various neurodegenerative conditions. When comparing paired cNfL and bNfL, they found a strong correlation for Simoa and ECL, but weaker for ELISA (Figure 1). They showed that cNfL were well correlated between technologies, whereas bNfL levels were only similar between ECL assay and Simoa, but not between ELISA and ECL, or and ELISA-Simoa (Figure 2). Similar findings were found by Gisslén et al. (2016). Generally, is it expected to find higher NfL levels when using Simoa, given its ultra-sensitivity. All together, these findings highlight the need of calibration and validation of cut-off levels across technologies (Kuhle et al., 2016a; Varhaug et al., 2019). Overall, Simoa is the preferred bNfL assay due to its low detection limit, simple and fast sampling, storage and handling, and feasibility of longitudinal sampling. Ongoing projects, led by Siemens Healthineers, are developing bNfL immunoassays using the Quanterix NfL antibodies. After reporting high correlation of bNfL levels between Simoa and the assay, they plan to adapt the Simoa assay onto a routine analyzer platform, which will accelerate the availability of NfL tests for patients around the world (Didner, 2019).

## NfL in CSF and Blood

**FIGURE 3:**
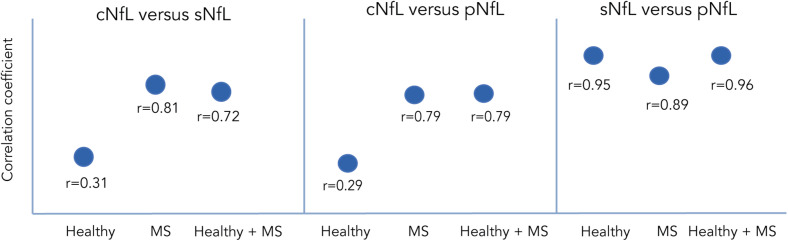
Correlation of NfL between CSF, serum and plasma in different population as reported by Harp et al. (2019), Hendricks et al. (2019), and Sejbaek et al. (2019). Correlation coefficients between NfL measured in CSF vs serum or vs plasma across different populations.

High variability between CSF and serum NfL (sNfL) levels have been found in pwMS (Figure 3). Harp and colleagues used the Simoa platform to compare levels of NfL in plasma, serum, and CSF samples obtained from the same individual, with and without brain pathology. Their results showed a strong correlation between serum and plasma NfL levels within the same person, but a weaker correlation between CSF and serum or plasma levels (Harp et al., 2019). Hendricks et al. (2019) analyzed NfL levels in 112 MS individuals and found a good correlation between CSF, serum, and plasma levels. Other group analyzed NfL levels in the CSF, plasma and serum of 52 untreated RRMS individuals, 23 healthy controls, and 52 MS individuals treated with placebo, matched by age, sex, and NfL (Sejbaek et al., 2019). They found that NfL levels were approximately 200 times higher in the CSF compared to plasma or serum. Additionally, even though the plasma and serum levels were highly correlated, plasma levels were 23% lower than in serum. Similar findings have been found in murine models of neurodegeneration (Gaiottino et al., 2013; Bacioglu et al., 2016; Disanto et al., 2017; Bianchi et al., 2019). Other authors examining treatment effects showed that both CSF and sNfL levels decrease with DMTs (Piehl et al., 2018; Sejbaek et al., 2019) suggested that serum levels might be more useful than plasma levels when evaluating treatment effects, given that sNfL levels show a relatively greater reduction compared to plasma levels.

It has been hypothesized that the differences between CSF, plasma/serum are probably due to the fact that the CSF compartment is closer to the damaged site, and to the integrity of the blood brain barrier, whilst concurrent peripheral inflammatory or infectious processes might affect the increase of blood NfL levels (Bacioglu et al., 2016; Sejbaek et al., 2019; Dalla Costa et al., 2019b). Despite the high variability found between compartments, most evidence seems to suggest there is a good correlation, which will likely shift the balance toward blood sampling given its practical advantages.

## Determining What Is Abnormal

A key point to validate NfL levels as surrogates of disease activity is establishing optimal and sensitive cut-off values. Currently, there is large heterogeneity on optimal values to establish what is pathological, to estimate the risk of conversion from RIS/CIS to clinically definite MS, to accurately predict disease progression, and to determine adequate treatment response (i.e., what would be considered a significant drop in NfL to reflect treatment response). Furthermore, gender and age stratification are lacking. Generally, cNfL cut-off levels range between 400–1,000 ng/l (Arrambide et al., 2016; Novakova et al., 2017b; Gaetani et al., 2019a), and corresponding sNfL cut-off levels between 3–30 ng/L (Norgren et al., 2004; Håkansson et al., 2018; Dalla Costa et al., 2019a; Thebault et al., 2020). Others have instead used percentiles across different ages (Disanto et al., 2017). More recently, authors have been calling for longitudinal measurements of sNfL rather than absolute cut-off values (Bittner et al., 2020; Ferraro et al., 2020; Häring et al., 2020).

This variability reflects the lack of consensus on measuring techniques between centres, no fixed criteria for setting abnormal values, and in determining the rate or percent change that would be clinically meaningful over time.

## Utility of NfL Measurement in Clinical Practice

Monitoring and predicting disease activity and treatment response is crucial for the individualized management of pwMS (Kalincik et al., 2017). MRI is a reliable tool to assess therapeutic monitoring, however, it is costly and is not always available owing to geographic reasons or lack of local resources. Recent studies have examined the effects of DMTs on NfL levels and evaluated the potential role as a therapy monitoring tool (Table 1). Kuhle et al. (2019a) measured sNfL in individuals with RRMS that were part of two ongoing clinical trials. Those receiving fingolimod had lower NfL levels throughout the course of the disease compared to those receiving IFNβ1a, which is congruent with clinical observations. Likewise, a multicenter Swedish study showed that both cNfL and sNfL levels correlated with DMT response, regardless of whether they were receiving DMTs or not (Novakova et al., 2017b). They observed that NfL levels were reduced in those initiating DMT for the first time or after switching from first- to second line DMTs. Similar results were found in studies analyzing the effect of dimethyl fumarate in individuals without previous therapy (Sejbaek et al., 2019), individuals treated with natalizumab (Gunnarsson et al., 2011) or alemtuzumab (Hyun et al., 2020), and individuals switching from IFN or glatiramer acetate to rituximab (de Flon et al., 2016), or from injectable therapies to fingolimod (Piehl et al., 2018). Interestingly, Dalla Costa et al. (2019a) analyzed the role of NfL as markers of progressive multifocal leukoencephalopathy (PML) secondary to natalizumab therapy. They showed that sNfL had a tenfold increase with PML onset, which was higher than those having an MS relapse.

**TABLE 1:** Reported reduction in NfL levels after treatment with DMTs.

DMTs	Reduction at last follow up
Natalizumab	37%, *p* = 0.03 (Christensen et al., 2014)
	20%, *p* < 0.001 (Kapoor et al., 2018)
	30%, *p* < 0.001 (Gunnarsson et al., 2011)
Fingolimod vs IFNβ1a	38%, *p* < 0.001 (Kuhle et al., 2019a)
Alemtuzumab and disease activity	DMT associated with no disease activity *p* < 0.001 (Hyun et al., 2020)
IFN or glatiramer acetate to rituximab	21%, *p* = 0.01 (de Flon et al., 2016)
Injectable therapies to fingolimod	33%, *p* < 0.001 (Piehl et al., 2018)
Ocrelizumab vs placebo	43%, *p* < 0.001 (Bar -Or et al., 2019)
Ibudilast vs placebo	no difference (Fox et al., 2021)

Individualized management is a key goal in MS management. Current treatment strategies are based on clinical and radiological findings; however, these often fail to capture the full extent of the disease and have a limited correlation with disability and prognosis. With the recent findings on the correlation of NfL levels and disease activity, disability and therapy monitoring, the next step is to examine whether including NfL measurements into day-to-day clinical practice is beneficial. A recent observational study examined the role of NfL measurement in treatment decisions and as a surrogate marker of clinical or radiological activity (Reyes et al., 2020). They found that NfL levels were closely associated with clinical and radiological activity, and in a significant proportion of individuals with PMS, elevated NfL was the only evidence of disease activity used in the treatment decision-making process. In line with the available evidence, individuals with elevated NfL levels were more likely to have treatment escalation as a means to reduce axonal damage and neurodegeneration. Importantly, they emphasized the need of age-related cut-offs and predefined time intervals for measurements as indispensable prior to introducing NfL measurements in clinical decision making.

## Role of NfL in Research

To date, more than 2000 MS clinical trials are active^1^. Of these, more than 150 correspond to phase 2 trials, of which one third are testing new drugs. A large percentage of these trials use MRI measures to evaluate outcomes. Even though there is substantial evidence on the association between MRI findings, disease progression and treatment response, subclinical activity and neuroaxonal damage markers cannot be fully evaluated with available neuroimaging techniques (Gajofatto et al., 2013). In addition, long-term (i.e., 24 months or more) MRI-based endpoints do not correlate as well as short term imaging endpoints, and therefore frequent scans are usually needed. Repeated scans increase costs, are burdensome for many individuals, and do not usually cover the spinal cord. Given the advantages of NfL measurement and the good correlation it has shown with MRI findings of disease activity and treatment response, it has gained significant attention as a potential end point marker for clinical trials (Sormani et al., 2019; Kuhle et al., 2019a). In addition, the findings from the nested case-control study of United States military personnel by Bjornevik et al. (2020) suggest that NfL levels in asymptomatic individuals could be used to select candidates that would benefit from disease prevention trials.

## The Known Unknowns

### How Much NfL From Peripheral Nerve Disease Will Affect Serum/CSF NfL?

The effect of peripheral nerve disease on NfL levels has not been widely studied but will be highly relevant when incorporating NfL measurements in the clinical management of pwMS. Mariotto et al. (2018) studied a cohort of 25 individuals with acquired peripheral neurological disease and observed a significant increase between cNfL and sNfL and disease activity, severity and outcome, although only sNFL remain significant. Interestingly, they noticed a correlation between NfL only in individuals with possible brain-nerve barrier damage, suggesting that disrupted brain-nerve barrier could contribute to the increase in cNfL seen in patients with peripheral nerve disorders. Similar results were found in a study that included individuals with Charcot Marie Tooth disease (Sandelius et al., 2018), vascular neuropathy (Bischof et al., 2018), progressive axonal sensorimotor polyneuropathy (Louwsma et al., 2020), and prediabetic neuropathy (Celikbilek et al., 2014). More studies will be needed to characterize the impact of peripheral nerve disease in NfL levels in individuals with concomitant CNS disease.

### Can Anti-NfL Antibodies Reduce the Circulating NfL Levels?

The generation of antibodies against neuronal antigens, such as neurofilament light, has been reported (Bartoš et al., 2007). There is some evidence on the relationship between anti-NfL antibodies and circulating NfL levels. Fialová et al. (2013) demonstrated increased NfL levels and intrathecal synthesis of anti-NfL and anti-NfH autoantibodies in early MS, but not in those with long standing disease. They suggested that this observation was in line with the clinical observation that autoimmune mechanisms predominate at initial phases of the disease. Interestingly, they also observed an association between anti-NfL levels and early conversion to clinically definite MS in individuals with CIS. They concluded that CSF anti-NfL antibodies and CSF anti-NfL/serum anti-NfL antibody ratio could help differentiate individuals with CIS with a higher risk of conversion. Moreover, Silber et al. (2002) observed that anti-Nfl and anti-NfH correlated with disease duration and EDSS, and Amor et al. (2014) observed a reduction in anti-NfL levels after natalizumab therapy. Despite these findings, the clinical usefulness of anti-Nf measurement is not straightforward. Other groups have reported presence of anti-NfM antibodies in individuals with non-immune neurological disorders, such as migraine and chronic fatigue syndrome (Bartoš et al., 2007). Also, discrepancy between CSF and serum levels for these antibodies have been documented (Ehling et al., 2004; Bartoš et al., 2007), which limits its utility as an isolated measurement (Dubuisson et al., 2017). Further studies are required to draw solid conclusions about the impact of anti-Nf antibodies on circulating NfL and their clinical utility.

### Other Biomarkers, Where do They Stand?

Even though there is a higher volume of evidence and increased clinical and research interest in NfL as a biomarker in MS, there are other biomarkers that have been examined independently and in relation to NfL.

Oligoclonal bands are detected in the CSF of about 95% of pwMS and are considered the best diagnostic element supportive of MS diagnosis (Deisenhammer et al., 2019). CSF OCBs are produced by B cells and plasma cells and can be found in other neuroinflammatory diseases. OCBs positivity, independent of MRI lesion load, strongly predicts conversion from clinically isolated syndromes (CIS) to CDMS (Dobson and Giovannoni, 2019). The predicate risk increases significantly (adjusted hazard ratio 11.3 (6.7–19.3) when OCBs are found together with 10 or more lesions on MRI (Ignacio et al., 2010; Tintore et al., 2015). Similarly, studies examining the predictive role of OCBs and cNfL have shown that individuals with RIS presenting with positive OCBs and/or high levels of cNfL have a shorter time to conversion to CIS and MS compared to those with negative OCB and/or low cNfL levels (Matute-Blanch et al., 2018). Interestingly, the association between cNfL and time to conversion was restricted to individuals older than 37 years, whereas the one with OCBs was present regardless of age. Among pwMS, a low number of OCBs at diagnosis may be associated with a better prognosis and treatment response (Avasarala et al., 2001; Joseph et al., 2009), however, no clear prognostic value has been established (Becker et al., 2015).

Osteopontin (OPN) is an extracellular matrix protein widely expressed in immune cells and involved in T-mediated inflammatory response (Brown, 2012). OPN expression has been found in MS lesions (Chabas et al., 2001) and specific OPN genotypes have been associated with an increased risk of developing MS (Chiocchetti et al., 2005). Previous studies have shown that OPN levels are increased in secondary progressive MS (SPMS; Comabella et al., 2005; Romme Christensen et al., 2013; Shimizu et al., 2013), PPMS and RRMS (Vogt et al., 2003; Romme Christensen et al., 2013). They have also been associated with cognitive impairment and treatment response (Iaffaldano et al., 2014). A recent meta-analysis concluded that elevated levels of OPN in CSF and in the peripheral blood of pwMS are suggestive of active inflammation (Agah et al., 2018). A study by Tortorella and colleagues found that OPN was inversely correlated with the volume of the corpus callosum, whereas NfL was associated with gray matter volume in a cohort of individuals with CIS (Direnzo et al., 2015). They suggested that cNfL and OPN levels during CIS might reflect different patterns of early neurodegeneration. A study examining the stepwise predictive value of 18 biomarkers including cNfL and cOPN and MRI lesion load concluded that baseline cNfL combined with OPN and CLL2 correctly predicted the clinical activity status of 91% of the individuals with CIS or MS. In contrast, cOPN + cNfL, cNfl alone, T2 lesion load + cNfL or alone, showed lower percentages of correct prediction (86, 83, 81, and 71%, respectively; Håkansson et al., 2017).

C-X-C motif chemokine-13 (CXCL13) is a B cell chemoattractant that has been shown to be involved in the recruitment of B cells into the CNS during neuroinflammatory conditions (Cui et al., 2020). Unlike NFL, CXCL13 is not commonly produced in the absence of neuroinflammatory conditions. Recent evidence indicates that CXCL13 is associated with prognosis and disease activity, and is reduced with corticosteroids, fingolimod, natalizumab and B-cell depletion therapies (Lycke and Zetterberg, 2017; Matute-Blanch et al., 2017). CXCL13 levels alone or in combination with NFL might also predict CIS conversion to MS (Brettschneider et al., 2010). Interestingly, a recent study examining the clinical utility of combined cNfL and CXCL13 measurements concluded that these biomarkers continue to be evaluated in individuals with no clinical or radiological activity, which could complement the assessment of disease activity in pwMS (Novakova et al., 2020). Remarkably, DiSano and collegues recently showed that CXCL13 combined with NfL had excellent sensitivity (100%), specificity (72%), positive predictive value (71%), and negative predictive value (100%) compared to either CXCL13 or NfL alone to predictive future disease activity (DiSano et al., 2020). Additionally, they showed that CXCL13 + NfL had better predictive value compared to NfL + OCBs, CXCL13 + OCBs, and CXCL13 + OCBs + NfL to predictive disease activity in pwMS.

Chitinase-3-like protein 1 (CHI3L1) is a glycosidase secreted by monocytes, astrocytes and microglia and it is thought to modulate CNS inflammation. There is evidence that CHI3L1 levels are associated with CIS conversion to MS, disability progression (Cantó et al., 2011; Hinsinger et al., 2015; Håkansson et al., 2018; Huss et al., 2020), and presumably treatment response (Matute-Blanch et al., 2018). Similar to other biomarkers of neuroinflammation and tissue damage, CHI3L1 is not specific for MS and has been found to be elevated in cancer and rheumatoid arthritis (Tsuruha et al., 2002). A recent study examining the value of cNfL and CHI3L1 concluded that CHI3L1 levels were associated with spinal cord volume loss but not with brain gray matter atrophy. In contrast, cNfL was associated with brain but not with spinal cord volumes (Schneider et al., 2021). The combined measurement of CHI3L1 + cNfL could therefore provide complementary information on the location of atrophy in pwMS. Gil-Perotin et al. also provided evidence of the utility of CHI3L1 levels over NfL to discriminate different MS phenotypes (Gil-Perotin et al., 2019).

## Future Directions

### Standardization and Guidelines

There is now a large pool of data supporting the use of NfL in MS, directing it toward the adoption of NfL in clinical trial protocols (Kapoor et al., 2020). Evidence for use of NfL in routine clinical practice is also mounting suggesting that cNfL can complement established markers of disease activity to guide treatment strategies in MS (Reyes et al., 2020), adding further weight to the argument that NfL can be included in clinical guidelines. However, important gaps remain, particularly concerning validity, which stems from the lack (and need) of standardized values across the world. These include. standardization of NfL measurement techniques (i.e., sample collection and assay methods) and a well-defined diagnostic and prognostic cut-off levels, for both healthy individuals and MS.

Multicenter studies have reported a low variation of NfL and NfH measurements across sites and between assays (Oeckl et al., 2016; Kuhle et al., 2018), however, more is needed to evaluate variation across batches (Sharma et al., 2018). There is also a pressing need to establish ideal sampling time windows (e.g., timing with a relapse) and assessing their impact on the clinical predictive value of NfL levels in the long-term. This will enable comparisons across individuals and the optimization of resources, both in the clinical and research setting. Multicenter collaborations are therefore needed to address these gaps, with formulation of guidelines that address use and limitations of this test.

### Isobaric Tags and Dried Plasma Spots

Large scale biomarker studies and routine clinical measurement of biomarkers come at a great cost (Collinson, 2015). Isobaric tags and dried plasma spot (DPS) have emerged as potential measurement substrates that could reduce costs, processing times, and the need of specialized infrastructure. A recent study analyzed NfL levels in 17 individuals using DPS and Simoa (Lombardi et al., 2020). They observed a good discrimination of ALS from healthy controls, which was comparable to the discrimination obtained using sNfL measures. However, biological interaction with other blood components may interfere with quantitative and qualitative measurements. Leoni et al. (2019) observed good sensitivity of isobaric tags to proteins linked to ALS, including neurofilament light. To our knowledge, no studies have evaluated the use of DPS to measure NfL in MS.

## Conclusion

To date, diagnosis, management, and prognosis of MS relays on neuroimaging and clinical findings. However, the discovery of biomarkers such as NfL is turning the page toward a much desired and needed individualized medicine. NfL, a biological surrogate of CNS axonal damage, has consistently shown to reflect both clinical and subclinical changes in the activity and short-term burden of the disease. It has also proven to be an excellent indicator of treatment response and even as predictor of MS in presymptomatic individuals, which adds value to its utility as a clinical trial endpoint. The technological developments seen in the past years, and the ones yet to come, are widening the access to minimally invasive, fast, and low-cost measurements of bNfL levels. Additionally, this will permit larger and more longitudinal studies to be carried out, which in turn, will help determine and validate cut-off values according to the individual’s characteristics, current treatment status, and neurological comorbidities. In the upcoming years, we may see NfL being included in best clinical practice guidelines and it being routinely and longitudinally evaluates in MS. The inclusion of NfL measures into the clinical decision making will allow for more individualized and prompt management of MS, with accurate prognosis and optimized follow-up of patients presenting with MS and those with established MS.

## Author Contributions

CF-A contributed to the literature review and the preparation of the manuscript. CF-A, SR, GG, and SG contributed to the design of the manuscript and critically revised the manuscript for intellectual content. All authors contributed to the article and approved the submitted version.

## Conflict of Interest

The authors declare that the research was conducted in the absence of any commercial or financial relationships that could be construed as a potential conflict of interest.

## Footnotes

1 clinicaltrials.gov

## References

[B1] Agah E. Zardoui A. Saghazadeh A. Ahmadi M. Tafakhori A. Rezaei N. (2018). Osteopontin (OPN) as a CSF and blood biomarker for multiple sclerosis: a systematic review and meta-analysis. *PLoS One* 13:e0190252. 10.1371/journal.pone.0190252 29346446 PMC5773083

[B2] Aktas O. Renner A. Huss A. Filser M. Baetge S. Stute N. (2020). Serum neurofilament light chain: no clear relation to cognition and neuropsychiatric symptoms in stable MS. *Neurol. Neuroimmunol. Neuroinflamm.* 7:e885. 10.1212/nxi.0000000000000885 32972970 PMC7673283

[B3] Alkhasova M. Sutton P. Pettigrew L. Guduru Z. Avasarala J. (2020). Neuromyelitis optica spectrum disorders misdiagnosed as multiple sclerosis: can current diagnostic guidelines separate the two diseases? (1934). *Neurology* 94:134.

[B4] Amor S. Van Der Star B. J. Bosca I. Raffel J. Gnanapavan S. Watchorn J. (2014). Neurofilament light antibodies in serum reflect response to natalizumab treatment in multiple sclerosis. *Mult. Scler. J.* 20 1355–1362. 10.1177/1352458514521887 24515731

[B5] Anderson V. Bentley E. Loveless S. Bianchi L. Harding K. E. Wynford-Thomas R. A. (2020). Serum neurofilament-light concentration and real-world outcome in MS. *J. Neurol. Sci.* 417:117079. 10.1016/j.jns.2020.117079 32781395

[B6] Arrambide G. Espejo C. Eixarch H. Villar L. M. Alvarez-Cermeño J. C. Picón C. (2016). Neurofilament light chain level is a weak risk factor for the development of MS. *Neurology* 87 1076–1084. 10.1212/WNL.0000000000003085 27521440 PMC5027802

[B7] Arrambide G. Espejo C. Tintore M. (2015). The only certain measure of the effectiveness of multiple sclerosis therapy is cerebrospinal neurofilament level - NO. *Mult. Scler.* 21 1240–1242. 10.1177/1352458515589774 26242692

[B8] Avasarala J. R. Cross A. H. Trotter J. L. (2001). Oligoclonal band number as a marker for prognosis in multiple sclerosis. *Arch. Neurol.* 58 2044–2045. 10.1001/archneur.58.12.2044 11735778

[B9] Axelsson M. Malmeström C. Gunnarsson M. Zetterberg H. Sundström P. Lycke J. (2014). Immunosuppressive therapy reduces axonal damage in progressive multiple sclerosis. *Mult. Scler.* 20 43–50. 10.1177/1352458513490544 23702432

[B10] Bacioglu M. Maia L. F. Preische O. Schelle J. Apel A. Kaeser S. A. (2016). Neurofilament light chain in blood and CSF as marker of disease progression in mouse models and in neurodegenerative diseases. *Neuron* 91 56–66. 10.1016/j.neuron.2016.05.018 27292537

[B11] Bar -Or A. Thane G. Harp C. Cross A. Hauser S. (2019). “Blood neurofilament light levels are lowered to a healthy donor range in patients with RMS and PPMS following ocrelizumab treatment,” in *ECTRIMS Online Library* (Berlin), 25–52.

[B12] Barro C. Benkert P. Disanto G. Tsagkas C. Amann M. Naegelin Y. (2018). Serum neurofilament as a predictor of disease worsening and brain and spinal cord atrophy in multiple sclerosis. *Brain* 141 2382–2391. 10.1093/brain/awy154 29860296

[B13] Bartoš A. Fialová L. Soukupová J. Kukal J. Malbohan I. Pit́ha J. (2007). Antibodies against light neurofilaments in multiple sclerosis patients. *Acta Neurol. Scand.* 116 100–107. 10.1111/j.1600-0404.2006.00794.x 17661795

[B14] Beart P. Robinson M. Rattray M. Maragakis N. J. (2017). “Erratum,” *Neurodegenerative Diseases. Advances in Neurobiology*, Vol. 15 eds Beart P. Robinson M. Rattray M. Maragakis N. (Cham: Springer), E1. 10.1007/978-3-319-57193-5 28905310

[B15] Becker M. Latarche C. Roman E. Debouverie M. Malaplate-Armand C. Guillemin F. (2015). No prognostic value of routine cerebrospinal fluid biomarkers in a population-based cohort of 407 multiple sclerosis patients. *BMC Neurol.* 15:79. 10.1186/s12883-015-0330-4 25966681 PMC4430897

[B16] Berard J. A. Smith A. M. Walker L. A. S. (2019). Predictive models of cognitive fatigue in multiple sclerosis. *Arch. Clin. Neuropsychol.* 34 31–38. 10.1093/arclin/acy014 29471423

[B17] Bergman J. Dring A. Zetterberg H. Blennow K. Norgren N. Gilthorpe J. (2016). Neurofilament light in CSF and serum is a sensitive marker for axonal white matter injury in MS. *Neurol. Neuroimmunol. NeuroInflamm.* 3:e271. 10.1212/NXI.0000000000000271 27536708 PMC4972001

[B18] Bhan A. Jacobsen C. Myhr K. M. Dalen I. Lode K. Farbu E. (2018). Neurofilaments and 10-year follow-up in multiple sclerosis. *Mult. Scler. J.* 24 1301–1307. 10.1177/1352458518782005 30066611

[B19] Bianchi L. Baker D. Giovannoni G. Marta M. (2019). “Neurofilament light chain levels in cerebrospinal fluid and serum of a longitudinal cohort of people with multiple sclerosis on disease modifying drugs,” in *ECTRIMS Online Library* (Stockholm), 1339.

[B20] Bischof A. Manigold T. Barro C. Heijnen I. Berger C. T. Derfuss T. (2018). Serum neurofilament light chain: a biomarker of neuronal injury in vasculitic neuropathy. *Ann. Rheum. Dis.* 77 1093–1094. 10.1136/annrheumdis-2017-212045 28743789

[B21] Bittner S. Steffen F. Uphaus T. Muthuraman M. Fleischer V. Salmen A. (2020). Clinical implications of serum neurofilament in newly diagnosed MS patients: a longitudinal multicentre cohort study. *EBioMedicine* 56:102807. 10.1016/j.ebiom.2020.102807 32460167 PMC7251380

[B22] Bjartmar C. Trapp B. D. (2001). Axonal and neuronal degeneration in multiple sclerosis: mechanisms and functional consequences. *Curr. Opin. Neurol.* 14 271–278. 10.1097/00019052-200106000-00003 11371748

[B23] Bjornevik K. Munger K. L. Cortese M. Barro C. Healy B. C. Niebuhr D. W. (2020). Serum neurofilament light chain levels in patients with presymptomatic multiple sclerosis. *JAMA Neurol.* 77 58–64. 10.1001/jamaneurol.2019.3238 31515562 PMC6745051

[B24] Brettschneider J. Czerwoniak A. Senel M. Fang L. Kassubek J. Pinkhardt E. (2010). The chemokine CXCL13 is a prognostic marker in clinically isolated syndrome (CIS). *PLoS One* 5:e11986. 10.1371/journal.pone.0011986 20700489 PMC2916843

[B25] Bridel C. Van Wieringen W. N. Zetterberg H. Tijms B. M. Teunissen C. E. Alvarez-Cermeño J. C. (2019). Diagnostic value of cerebrospinal fluid neurofilament light protein in neurology: a systematic review and meta-analysis. *JAMA Neurol.* 76 1035–1048. 10.1001/jamaneurol.2019.1534 31206160 PMC6580449

[B26] Brown A. (2012). Osteopontin: a key link between immunity, inflammation and the central nervous system. *Transl. Neurosci.* 3 288–293. 10.2478/s13380-012-0028-7 23565338 PMC3616337

[B27] Cantó E. Barro C. Zhao C. Caillier S. J. Michalak Z. Bove R. (2019). Association between serum neurofilament light chain levels and long-term disease course among patients with multiple sclerosis followed up for 12 years. *JAMA Neurol.* 76 1359–1366. 10.1001/jamaneurol.2019.2137 31403661 PMC6692664

[B28] Cantó E. Reverter F. Matesanz F. (2011). Chitinase 3-like 1 plasma levels are increased in patients with progressive forms of multiple sclerosis. *Mult. Scler.* 18 983–990. 10.1177/1352458511433063 22183936

[B29] Celikbilek A. Tanik N. Sabah S. Borekci E. Akyol L. Ak H. (2014). Elevated neurofilament light chain (NFL) mRNA levels in prediabetic peripheral neuropathy. *Mol. Biol. Rep.* 41 4017–4022. 10.1007/s11033-014-3270-y 24733614

[B30] Chabas D. Baranzini S. E. Mitchell D. Bernard C. C. A. Rittling S. R. Denhardt D. T. (2001). The influence of the proinflammatory cytokine, osteopontin, on autoimmue demyelinating desease. *Science* 294 1731–1735. 10.1126/science.1062960 11721059

[B31] Chiocchetti A. Comi C. Indelicato M. Castelli L. Mesturini R. Bensi T. (2005). Osteopontin gene haplotypes correlate with multiple sclerosis development and progression. *J. Neuroimmunol.* 163 172–178. 10.1016/j.jneuroim.2005.02.020 15885319

[B32] Chitnis T. Gonzalez C. Healy B. C. Saxena S. Rosso M. Barro C. (2018). Neurofilament light chain serum levels correlate with 10-year MRI outcomes in multiple sclerosis. *Ann. Clin. Transl. Neurol.* 5 1478–1491. 10.1002/acn3.638 30564615 PMC6292183

[B33] Christensen J. R. Ratzer R. Börnsen L. Lyksborg M. Garde E. Dyrby T. B. (2014). Natalizumab in progressive MS: results of an open-label, phase 2A, proof-of-concept trial. *Neurology* 82 1499–1507. 10.1212/WNL.0000000000000361 24682973

[B34] Collinson P. (2015). Evidence and cost effectiveness requirements for recommending new biomarkers. *EJIFCC* 26 183–189. 27683493 PMC4975302

[B35] Comabella M. Pericot I. Goertsches R. Nos C. Castillo M. Blas Navarro J. (2005). Plasma osteopontin levels in multiple sclerosis. *J. Neuroimmunol.* 158 231–239. 10.1016/j.jneuroim.2004.09.004 15589058

[B36] Cui L. Y. Chu S. F. Chen N. H. (2020). The role of chemokines and chemokine receptors in multiple sclerosis. *Int. Immunopharmacol.* 83:106314. 10.1016/j.intimp.2020.106314 32197226 PMC7156228

[B37] Dalla Costa G. Martinelli V. Moiola L. Sangalli F. Colombo B. Finardi A. (2019a). Serum neurofilaments increase at progressive multifocal leukoencephalopathy onset in natalizumab-treated multiple sclerosis patients. *Ann. Neurol.* 85 606–610. 10.1002/ana.25437 30761586

[B38] Dalla Costa G. Martinelli V. Sangalli F. Moiola L. Colombo B. Radaelli M. (2019b). Prognostic value of serum neurofilaments in patients with clinically isolated syndromes. *Neurology* 92 E733–E741. 10.1212/WNL.0000000000006902 30635483 PMC6382362

[B39] Damasceno A. Dias-Carneiro R. P. C. Moraes A. S. Boldrini V. O. Quintiliano R. P. S. da Silva V. A. (2019). Clinical and MRI correlates of CSF neurofilament light chain levels in relapsing and progressive MS. *Mult. Scler. Relat. Disord.* 30 149–153. 10.1016/j.msard.2019.02.004 30772673

[B40] de Flon P. Gunnarsson M. Laurell K. Söderström L. Birgander R. Lindqvist T. (2016). Reduced inflammation in relapsing-remitting multiple sclerosis after therapy switch to rituximab. *Neurology* 87 141–147. 10.1212/WNL.0000000000002832 27316241

[B41] de Stefano N. Narayanan S. Francis S. J. Smith S. Mortilla M. Carmela Tartaglia M. (2002). Diffuse axonal and tissue injury in patients with multiple sclerosis with low cerebral lesion load and no disability. *Arch. Neurol.* 59 1565–1571. 10.1001/archneur.59.10.1565 12374493

[B42] de Wolf F. Ghanbari M. Licher S. McRae-McKee K. Gras L. Weverling G. J. (2020). Plasma tau, neurofilament light chain and amyloid-b levels and risk of dementia; a population-based cohort study. *Brain* 143 1220–1232. 10.1093/brain/awaa054 32206776 PMC7174054

[B43] Deisenhammer F. Zetterberg H. Fitzner B. Zettl U. K. (2019). The cerebrospinal fluid in multiple sclerosis. *Front. Immunol.* 10:726. 10.3389/fimmu.2019.00726 31031747 PMC6473053

[B44] Didner S. (2019). *Siemens Healthineers Enters into License and Supply Arrangement with Quanterix for Access to Neurofilament Light (Nf-L) Antibodies to Develop Nf-L Assays.* Billerica, MA: Quanterix.

[B45] Direnzo V. Tortorella C. Zoccolella S. Ruggieri M. Mastrapasqua M. Paolicelli D. (2015). Cerebrospinal fluid osteopontin and neurofilament levels mark different patterns of brain atrophy in clinically isolated syndrome (P5.218). *Neurology* 84:14.

[B46] DiSano K. D. Gilli F. Pachner A. R. (2020). Intrathecally produced CXCL13: a predictive biomarker in multiple sclerosis. *Mult. Scler. J. Exp. Transl. Clin.* 6:205521732098139. 10.1177/2055217320981396 33403120 PMC7747124

[B47] Disanto G. Adiutori R. Dobson R. Martinelli V. Costa G. D. Runia T. (2016). Serum neurofilament light chain levels are increased in patients with a clinically isolated syndrome. *J. Neurol. Neurosurg. Psychiatry* 87 126–129. 10.1136/jnnp-2014-309690 25716934

[B48] Disanto G. Barro C. Benkert P. Naegelin Y. Schädelin S. Giardiello A. (2017). Serum neurofilament light: a biomarker of neuronal damage in multiple sclerosis. *Ann. Neurol.* 81 857–870. 10.1002/ana.24954 28512753 PMC5519945

[B49] Dobson R. Giovannoni G. (2019). Multiple sclerosis – a review. *Eur. J. Neurol.* 26 27–40. 10.1111/ene.13819 30300457

[B50] Domingues R. B. Fernandes G. B. P. Leite F. B. V. D. M. Senne C. (2019). Neurofilament light chain in the assessment of patients with multiple sclerosis. *Arq. Neuropsiquiatr.* 77 436–441. 10.1590/0004-282x20190060 31314847

[B51] Dubuisson N. Puentes F. Giovannoni G. Gnanapavan S. (2017). Science is 1% inspiration and 99% biomarkers. *Mult. Scler.* 23 1442–1452. 10.1177/1352458517709362 28537780

[B52] Ehling R. Lutterotti A. Wanschitz J. Khalil M. Gneiss C. Deisenhammer F. (2004). Increased frequencies of serum antibodies to neurofilament light in patients with primary chronic progressive multiple sclerosis. *Mult. Scler.* 10 601–606. 10.1191/1352458504ms1100oa 15584481

[B53] Ehrenberg A. J. Khatun A. Coomans E. Betts M. J. Capraro F. Thijssen E. H. (2020). Relevance of biomarkers across different neurodegenerative. *Alzheimers Res. Ther.* 12:56. 10.1186/s13195-020-00601-w 32404143 PMC7222479

[B54] Ferraro D. Guicciardi C. de Biasi S. Pinti M. Bedin R. Camera V. (2020). Plasma neurofilaments correlate with disability in progressive multiple sclerosis patients. *Acta Neurol. Scand.* 141 16–21. 10.1111/ane.13152 31350854

[B55] Fialová L. Bartos A. Švarcová J. Zimova D. Kotoucova J. Malbohan I. (2013). Serum and cerebrospinal fluid light neurofilaments and antibodies against them in clinically isolated syndrome and multiple sclerosis. *J. Neuroimmunol.* 262 113–120. 10.1016/j.jneuroim.2013.06.010 23870535

[B56] Filippi M. Bozzali M. Rovaris M. Gonen O. Kesavadas C. Ghezzi A. (2003). Evidence for widespread axonal damage at the earliest clinical stage of multiple sclerosis. *Brain* 126 433–437. 10.1093/brain/awg038 12538409

[B57] Fox R. J. Raska P. Barro C. Karafa M. Konig V. Bermel R. A. (2021). Neurofilament light chain in a phase 2 clinical trial of ibudilast in progressive multiple sclerosis. *Mult. Scler. J.* 135245852098695. 10.1177/1352458520986956 33635141 PMC8387506

[B58] Fujihara K. (2019). Neuromyelitis optica spectrum disorders: still evolving and broadening. *Curr. Opin. Neurol.* 32 385–394. 10.1097/WCO.0000000000000694 30893099 PMC6522202

[B59] Furby J. Hayton T. Anderson V. Altmann D. Brenner R. Chataway J. (2008). Magnetic resonance imaging measures of brain and spinal cord atrophy correlate with clinical impairment in secondary progressive multiple sclerosis. *Mult. Scler.* 14 1068–1075. 10.1177/1352458508093617 18632782

[B60] Fyfe I. (2018). Multiple sclerosis: CSF markers predict progression from radiologically isolated syndrome. *Nat. Rev. Neurol.* 14:194. 10.1038/nrneurol.2018.26 29497156

[B61] Gadea M. Martínez-Bisbal M. C. Marti-Bonmatí L. Espert R. Casanova B. Coret F. (2004). Spectroscopic axonal damage of the right locus coeruleus relates to selective attention impairment in early stage relapsing-remitting multiple sclerosis. *Brain* 127 89–98. 10.1093/brain/awh002 14506072

[B62] Gaetani L. Blennow K. Calabresi P. Di Filippo M. Parnetti L. Zetterberg H. (2019a). Neurofilament light chain as a biomarker in neurological disorders. *J. Neurol. Neurosurg. Psychiatry* 90 870–881. 10.1136/jnnp-2018-320106 30967444

[B63] Gaetani L. Eusebi P. Mancini A. Gentili L. Borrelli A. Parnetti L. (2019b). Cerebrospinal fluid neurofilament light chain predicts disease activity after the first demyelinating event suggestive of multiple sclerosis. *Mult. Scler. Relat. Disord.* 35 228–232. 10.1016/j.msard.2019.07.025 31404762

[B64] Gaetani L. Höglund K. Parnetti L. Pujol-Calderon F. Becker B. Eusebi P. (2018). A new enzyme-linked immunosorbent assay for neurofilament light in cerebrospinal fluid: analytical validation and clinical evaluation. *Alzheimers Res. Ther.* 10:8. 10.1186/s13195-018-0339-1 29370869 PMC6389166

[B65] Gaetani L. Salvadori N. Lisetti V. Eusebi P. Mancini A. Gentili L. (2019c). Cerebrospinal fluid neurofilament light chain tracks cognitive impairment in multiple sclerosis. *J. Neurol.* 266 2157–2163. 10.1007/s00415-019-09398-7 31129709

[B66] Gafson A. Craner M. J. Matthews P. M. (2017). Personalised medicine for multiple sclerosis care. *Mult. Scler.* 23 362–369. 10.1177/1352458516672017 27672137

[B67] Gaiottino J. Norgren N. Dobson R. Topping J. Nissim A. Malaspina A. (2013). Increased neurofilament light chain blood levels in neurodegenerative neurological diseases. *PLoS One* 8:75091. 10.1371/journal.pone.0075091 24073237 PMC3779219

[B68] Gajofatto A. Calabrese M. Benedetti M. D. Monaco S. (2013). Clinical, MRI, and CSF markers of disability progression in multiple sclerosis. *Dis. Markers* 35 687–699. 10.1155/2013/484959 24324285 PMC3842089

[B69] Gasperini C. Prosperini L. Tintoré M. Sormani M. P. Filippi M. Rio J. (2019). Unraveling treatment response in multiple sclerosis: a clinical and MRI challenge. *Neurology* 92 180–192. 10.1212/WNL.0000000000006810 30587516 PMC6345120

[B70] Gil-Perotin S. Castillo-Villalba J. Cubas-Nuñez L. Gasque R. Hervas D. Gomez-Mateu J. (2019). Combined cerebrospinal fluid neurofilament light chain protein and chitinase-3 like-1 levels in defining disease course and prognosis in multiple sclerosis. *Front. Neurol.* 10:1008. 10.3389/fneur.2019.01008 31608004 PMC6768010

[B71] Giovannoni G. (2006). Multiple sclerosis cerebrospinal fluid biomarkers. *Dis. Markers* 22 187–196. 10.1155/2006/509476 17124340 PMC3851677

[B72] Gisslén M. Price R. W. Andreasson U. Norgren N. Nilsson S. Hagberg L. (2016). Plasma concentration of the neurofilament light protein (NFL) is a biomarker of CNS injury in HIV infection: a cross-sectional study. *EBioMedicine* 3 135–140. 10.1016/j.ebiom.2015.11.036 26870824 PMC4739412

[B73] Gnanapavan S. Giovannoni G. (2015). Developing biomarkers for MS. *Curr. Top. Behav. Neurosci.* 26 179–194. 10.1007/7854_2014_362 25502545

[B74] Gnanapavan S. Grant D. Morant S. Furby J. Hayton T. Teunissen C. E. (2013). Biomarker report from the phase II lamotrigine trial in secondary progressive MS - neurofilament as a surrogate of disease progression. *PLoS One* 8:e70019. 10.1371/journal.pone.0070019 23936370 PMC3731296

[B75] Gunnarsson M. Malmeström C. Axelsson M. Sundström P. Dahle C. Vrethem M. (2011). Axonal damage in relapsing multiple sclerosis is markedly reduced by natalizumab. *Ann. Neurol.* 69 83–89. 10.1002/ana.22247 21280078

[B76] Håkansson I. Johansson L. Dahle C. Vrethem M. Ernerudh J. (2019). Fatigue scores correlate with other self-assessment data, but not with clinical and biomarker parameters, in CIS and RRMS. *Mult. Scler. Relat. Disord.* 36 101424. 10.1016/j.msard.2019.101424 31586802

[B77] Håkansson I. Tisell A. Cassel P. Blennow K. Zetterberg H. Lundberg P. (2017). Neurofilament light chain in cerebrospinal fluid and prediction of disease activity in clinically isolated syndrome and relapsing–remitting multiple sclerosis. *Eur. J. Neurol.* 24 703–712. 10.1111/ene.13274 28261960

[B78] Håkansson I. Tisell A. Cassel P. Blennow K. Zetterberg H. Lundberg P. (2018). Neurofilament levels, disease activity and brain volume during follow-up in multiple sclerosis. *J. Neuroinflamm.* 15:209. 10.1186/s12974-018-1249-7 30021640 PMC6052680

[B79] Häring D. A. Kropshofer H. Kappos L. Cohen J. A. Shah A. Meinert R. (2020). Long-term prognostic value of longitudinal measurements of blood neurofilament levels. *Neurol. Neuroimmunol. Neuroinflamm.* 7:e856. 10.1212/NXI.0000000000000856 32817406 PMC7428358

[B80] Harp C. T. Hendricks R. Fischer S. K. Brumm J. Herman A. H. (2019). Neurofilament light chain (NfL) levels in CSF, serum, and plasma of healthy donors using the Quanterix NfL advantage Kit^TM^ (P1.9-032). *Neurology* 92:15.

[B81] Hendricks R. Baker D. Brumm J. Davancaze T. Harp C. Herman A. (2019). Establishment of neurofilament light chain Simoa assay in cerebrospinal fluid and blood. *Bioanalysis* 11 1405–1418. 10.4155/bio-2019-0163 31401845

[B82] Hinsinger G. Galéotti N. Nabholz N. Urbach S. Rigau V. Demattei C. (2015). Chitinase 3-like proteins as diagnostic and prognostic biomarkers of multiple sclerosis. *Mult. Scler.* 21 1251–1261. 10.1177/1352458514561906 25698171

[B83] Huss A. Otto M. Senel M. Ludolph A. C. Abdelhak A. Tumani H. (2020). A score based on NfL and glial markers may differentiate between relapsing–remitting and progressive MS course. *Front. Neurol.* 11:608. 10.3389/fneur.2020.00608 32765393 PMC7378743

[B84] Hyun J. W. Kim Y. Kim G. Kim S. H. Kim H. J. (2020). Longitudinal analysis of serum neurofilament light chain: a potential therapeutic monitoring biomarker for multiple sclerosis. *Mult. Scler. J.* 26 659–667. 10.1177/1352458519840757 30912689

[B85] Iaffaldano P. Ruggieri M. Viterbo R. G. Mastrapasqua M. Trojano M. (2014). The improvement of cognitive functions is associated with a decrease of plasma Osteopontin levels in Natalizumab treated relapsing multiple sclerosis. *Brain. Behav. Immun.* 35 96–101. 10.1016/j.bbi.2013.08.009 23994630

[B86] Ignacio R. J. Liliana P. Edgardo C. (2010). Oligoclonal bands and MRI in clinically isolated syndromes: predicting conversion time to multiple sclerosis. *J. Neurol.* 257 1188–1191. 10.1007/s00415-010-5490-y 20157721

[B87] Joseph F. G. Hirst C. L. Pickersgill T. P. Ben-Shlomo Y. Robertson N. P. Scolding N. J. (2009). CSF oligoclonal band status informs prognosis in multiple sclerosis: a case control study of 100 patients. *J. Neurol. Neurosurg. Psychiatry* 80 292–296. 10.1136/jnnp.2008.150896 18829628

[B88] Kalincik T. Manouchehrinia A. Sobisek L. Jokubaitis V. Spelman T. Horakova D. (2017). Towards personalized therapy for multiple sclerosis: prediction of individual treatment response. *Brain* 140 2426–2443. 10.1093/brain/awx185 29050389

[B89] Kapoor R. Ho P. R. Campbell N. Chang I. Deykin A. Forrestal F. (2018). Effect of natalizumab on disease progression in secondary progressive multiple sclerosis (ASCEND): a phase 3, randomised, double-blind, placebo-controlled trial with an open-label extension. *Lancet Neurol.* 17, 405–415. 10.1016/S1474-4422(18)30069-3 29545067

[B90] Kapoor R. Smith K. E. Allegretta M. Arnold D. L. Carroll W. Comabella M. (2020). Serum neurofilament light as a biomarker in progressive multiple sclerosis. *Neurology* 95 436–444. 10.1212/WNL.0000000000010346 32675076 PMC7538221

[B91] Khalil M. Pirpamer L. Hofer E. Voortman M. M. Barro C. Leppert D. (2020). Serum neurofilament light levels in normal aging and their association with morphologic brain changes. *Nat. Commun.* 11:812. 10.1038/s41467-020-14612-6 32041951 PMC7010701

[B92] Khalil M. Teunissen C. E. Otto M. Piehl F. Sormani M. P. Gattringer T. (2018). Neurofilaments as biomarkers in neurological disorders. *Nat. Rev. Neurol.* 14 577–589. 10.1038/s41582-018-0058-z 30171200

[B93] Kim H. Lee E. J. Kim S. Choi L. K. Kim K. Kim H. W. (2020). Serum biomarkers in myelin oligodendrocyte glycoprotein antibody-associated disease. *Neurol. Neuroimmunol. Neuroinflamm.* 7:708. 10.1212/NXI.0000000000000708 32184342 PMC7136043

[B94] Kuhle J. Barro C. Andreasson U. Derfuss T. Lindberg R. Sandelius Å, et al. (2016a). Comparison of three analytical platforms for quantification of the neurofilament light chain in blood samples: ELISA, electrochemiluminescence immunoassay and Simoa. *Clin. Chem. Lab. Med.* 54 1655–1661. 10.1515/cclm-2015-1195 27071153

[B95] Kuhle J. Barro C. Disanto G. Mathias A. Soneson C. Bonnier G. (2016b). Serum neurofilament light chain in early relapsing remitting MS is increased and correlates with CSF levels and with MRI measures of disease severity. *Mult. Scler.* 22 1550–1559. 10.1177/1352458515623365 26754800

[B96] Kuhle J. Barro C. Hrusovsky K. Chang L. Jeromin A. Bridel C. (2018). “International multi-site analytical validation of the Simoa NF-light assay in human serum samples from multiple sclerosis patients,” in *ECTRIMS Online Library* (Berlin).

[B97] Kuhle J. Disanto G. Lorscheider J. Stites T. Chen Y. Dahlke F. (2015). Fingolimod and CSF neurofilament light chain levels in relapsing-remitting multiple sclerosis. *Neurology* 84 1639–1643. 10.1212/WNL.0000000000001491 25809304 PMC4409586

[B98] Kuhle J. Kropshofer H. Haering D. A. Kundu U. Meinert R. Barro C. (2019a). Blood neurofilament light chain as a biomarker of MS disease activity and treatment response. *Neurology* 92 E1007–E1015. 10.1212/WNL.0000000000007032 30737333 PMC6442011

[B99] Kuhle J. Plavina T. Barro C. Disanto G. Sangurdekar D. Singh C. M. (2019b). Neurofilament light levels are associated with long-term outcomes in multiple sclerosis. *Mult. Scler. J.* 26 1691–1699. 10.1177/1352458519885613 31680621 PMC7604552

[B100] Lassmann H. Brück W. Lucchinetti C. (2001). Heterogeneity of multiple sclerosis pathogenesis: implications for diagnosis and therapy. *Trends Mol. Med.* 7 115–121. 10.1016/S1471-4914(00)01909-2 11286782

[B101] Lee E. Lim Y. Kim S. Choi L. Kim H. Kim K. (2020). Clinical implication of serum biomarkers and patient age in inflammatory demyelinating diseases. *Ann. Clin. Transl. Neurol.* 7 992–1001. 10.1002/acn3.51070 32495489 PMC7317646

[B102] Lee J. Y. Taghian K. Petratos S. (2014). Axonal degeneration in multiple sclerosis: can we predict and prevent permanent disability? *Acta Neuropathol. Commun.* 2:97. 10.1186/s40478-014-0097-7 25159125 PMC4243718

[B103] Leoni E. Bremang M. Mitra V. Zubiri I. Jung S. Lu C. H. (2019). Combined tissue-fluid proteomics to unravel phenotypic variability in amyotrophic lateral sclerosis. *Sci. Rep.* 9:4478. 10.1038/s41598-019-40632-4 30872628 PMC6418138

[B104] Li D. Mielke M. M. (2019). An update on blood-based markers of Alzheimer’s disease using the SiMoA platform. *Neurol. Ther.* 8 73–82. 10.1007/s40120-019-00164-5 31833025 PMC6908531

[B105] Li D. K. B. Held U. Petkau J. Daumer M. Barkhof F. Fazekas F. (2006). MRI T2 lesion burden in multiple sclerosis: a plateauing relationship with clinical disability. *Neurology* 66 1384–1389. 10.1212/01.wnl.0000210506.00078.5c 16682671

[B106] Limberg M. Disanto G. Barro C. Kuhle J. (2015). Neurofilament light chain determination from peripheral blood samples. *Methods Mol. Biol.* 1304 93–98. 10.1007/7651_2015_206 25687302

[B107] Lombardi V. Carassiti D. Giovannoni G. Lu C. H. Adiutori R. Malaspina A. (2020). The potential of neurofilaments analysis using dry-blood and plasma spots. *Sci. Rep.* 10:97. 10.1038/s41598-019-54310-y 31919375 PMC6952412

[B108] Louwsma J. Brunger A. F. Bijzet J. Kroesen B. J. Roeloffzen W. W. H. Bischof A. (2020). Neurofilament light chain, a biomarker for polyneuropathy in systemic amyloidosis. *Amyloid* 28 50–55. 10.1080/13506129.2020.1815696 32883119

[B109] Lycke J. Zetterberg H. (2017). The role of blood and CSF biomarkers in the evaluation of new treatments against multiple sclerosis. *Expert Rev. Clin. Immunol.* 13 1143–1153. 10.1080/1744666X.2017.1400380 29090607

[B110] Lycke J. N. Karlsson J. E. Andersen O. Rosengren L. E. (1998). Neurofilament protein in cerebrospinal fluid: a potential marker of activity in multiple sclerosis. *J. Neurol. Neurosurg. Psychiatry* 64 402–404. 10.1136/jnnp.64.3.402 9527161 PMC2170011

[B111] Manouchehrinia A. Piehl F. Hillert J. Kuhle J. Alfredsson L. Olsson T. (2020a). Confounding effect of blood volume and body mass index on blood neurofilament light chain levels. *Ann. Clin. Transl. Neurol.* 7 139–143. 10.1002/acn3.50972 31893563 PMC6952306

[B112] Manouchehrinia A. Stridh P. Khademi M. Leppert D. Barro C. Michalak Z. (2020b). Plasma neurofilament light levels are associated with risk of disability in multiple sclerosis. *Neurology* 94 e2457–e2467. 10.1212/WNL.0000000000009571 32434867 PMC7455371

[B113] Marciniewicz E. Pokryszko-Dragan A. Podgórski P. Małyszczak K. Zimny A. Kołtowska A. (2019). Quantitative magnetic resonance assessment of brain atrophy related to selected aspects of disability in patients with multiple sclerosis: preliminary results. *Pol. J. Radiol.* 84 e171–e178. 10.5114/pjr.2019.84274 31481987 PMC6717938

[B114] Mariotto S. Farinazzo A. Magliozzi R. Alberti D. Monaco S. Ferrari S. (2018). Serum and cerebrospinal neurofilament light chain levels in patients with acquired peripheral neuropathies. *J. Peripher. Nerv. Syst.* 23 174–177. 10.1111/jns.12279 29974556

[B115] Mariotto S. Farinazzo S. Monaco S. Gajofatto A. Zanusso G. Schanda K. (2017). Serum neurofilament light chain in NMOSD and related disorders: comparison according to Aquaporin-4 and myelin oligodendrocyte glycoprotein antibodies status. *Mult. Scler. J. Exp. Transl. Clin.* 3:205521731774309. 10.1177/2055217317743098 29204292 PMC5703099

[B116] Mariotto S. Ferrari S. Gastaldi M. Franciotta D. Sechi E. Capra R. (2019). Neurofilament light chain serum levels reflect disease severity in MOG-Ab associated disorders. *J. Neurol. Neurosurg. Psychiatry* 90 1293–1296. 10.1136/jnnp-2018-320287 30952681

[B117] Martin S. J. McGlasson S. Hunt D. Overell J. (2019). Cerebrospinal fluid neurofilament light chain in multiple sclerosis and its subtypes: A meta-analysis of case-control studies. *J. Neurol. Neurosurg. Psychiatry* 90, 1059–1067. 10.1136/jnnp-2018-319190 31123141 PMC6820150

[B118] Martínez M. A. M. Olsson B. Bau L. Matas E. Calvo ÁC. Andreasson U. (2015). Glial and neuronal markers in cerebrospinal fluid predict progression in multiple sclerosis. *Mult. Scler. J.* 21 550–561. 10.1177/1352458514549397 25732842 PMC4390605

[B119] Matute-Blanch C. Montalban X. Comabella M. (2017). Multiple sclerosis, and other demyelinating and autoimmune inflammatory diseases of the central nervous system. *Handb. Clin. Neurol.* 146 3–20. 10.1016/B978-0-12-804279-3.00005-8 29110780

[B120] Matute-Blanch C. Villar L. M. Álvarez-Cermeño J. C. Rejdak K. Evdoshenko E. Makshakov G. (2018). Neurofilament light chain and oligoclonal bands are prognostic biomarkers in radiologically isolated syndrome. *Brain* 141 1085–1093. 10.1093/brain/awy021 29452342

[B121] Norgren N. Karlsson J. E. Rosengren L. Stigbrand T. (2002). Monoclonal antibodies selective for low molecular weight neurofilaments. *Hybrid. Hybridomics* 21 53–59. 10.1089/15368590252917647 11991817

[B122] Norgren N. Sundström P. Svenningsson A. Rosengren L. Stigbrand T. Gunnarsson M. (2004). Neurofilament and glial fibrillary acidic protein in multiple sclerosis. *Neurology* 63 1586–1590. 10.1212/01.WNL.0000142988.49341.D1 15534240

[B123] Novakova L. Axelsson M. Khademi M. Zetterberg H. Blennow K. Malmeström C. (2017a). Cerebrospinal fluid biomarkers of inflammation and degeneration as measures of fingolimod efficacy in multiple sclerosis. *Mult. Scler.* 23 62–71. 10.1177/1352458516639384 27003946

[B124] Novakova L. Axelsson M. Malmeström C. Imberg H. Elias O. Zetterberg H. (2018). Searching for neurodegeneration in multiple sclerosis at clinical onset: diagnostic value of biomarkers. *PLoS One* 13:e0194828. 10.1371/journal.pone.0194828 29614113 PMC5882126

[B125] Novakova L. Axelsson M. Malmeström C. Zetterberg H. Blennow K. Svenningsson A. (2020). NFL and CXCL13 may reveal disease activity in clinically and radiologically stable MS. *Mult. Scler. Relat. Disord.* 46:102463. 10.1016/j.msard.2020.102463 32862040

[B126] Novakova L. Zetterberg H. Sundström P. Axelsson M. Khademi M. Gunnarsson M. (2017b). Monitoring disease activity in multiple sclerosis using serum neurofilament light protein. *Neurology* 89 2230–2237. 10.1212/WNL.0000000000004683 29079686 PMC5705244

[B127] Oeckl P. Jardel C. Salachas F. Lamari F. Andersen P. M. Bowser R. (2016). Multicenter validation of CSF neurofilaments as diagnostic biomarkers for ALS. *Amyotroph. Lateral Scler. Front. Degener.* 17 404–413. 10.3109/21678421.2016.1167913 27415180

[B128] Pachner A. R. DiSano K. Royce D. B. Gilli F. (2019). Clinical utility of a molecular signature in inflammatory demyelinating disease. *Neurol. Neuroimmunol. NeuroInflamm.* 6:e520. 10.1212/NXI.0000000000000520 30568998 PMC6278854

[B129] Palotai M. Nazeri A. Cavallari M. Healy B. C. Glanz B. Gold S. M. (2019). History of fatigue in multiple sclerosis is associated with grey matter atrophy. *Sci. Rep.* 9:14781. 10.1038/s41598-019-51110-2 31611598 PMC6791855

[B130] Pascual A. M. Martínez-Bisbal M. C. Boscá I. Valero C. Coret F. Martínez-Granados B. (2007). Axonal loss is progressive and partly dissociated from lesion load in early multiple sclerosis. *Neurology* 69 63–67. 10.1212/01.wnl.0000265054.08610.12 17606882

[B131] Peng L. Bi C. Xia D. Mao L. Qian H. (2019). Increased cerebrospinal fluid neurofilament light chain in central nervous system inflammatory demyelinating disease. *Mult. Scler. Relat. Disord.* 30 123–128. 10.1016/j.msard.2019.02.009 30771578

[B132] Petrova N. Carassiti D. Altmann D. R. Baker D. Schmierer K. (2018). Axonal loss in the multiple sclerosis spinal cord revisited. *Brain Pathol.* 28 334–348. 10.1111/bpa.12516 28401686 PMC8028682

[B133] Petzold A. (2015). The prognostic value of CSF neurofilaments in multiple sclerosis at 15-year follow-up. *J. Neurol. Neurosurg. Psychiatry* 86 1388–1390. 10.1136/jnnp-2014-309827 25616604

[B134] Petzold A. Steenwijk M. D. Eikelenboom J. M. Wattjes M. P. Uitdehaag B. M. J. (2016). Elevated CSF neurofilament proteins predict brain atrophy: a 15-year follow-up study. *Mult. Scler.* 22 1154–1162. 10.1177/1352458516645206 27207456

[B135] Piehl F. Kockum I. Khademi M. Blennow K. Lycke J. Zetterberg H. (2018). Plasma neurofilament light chain levels in patients with MS switching from injectable therapies to fingolimod. *Mult. Scler. J.* 24 1046–1054. 10.1177/1352458517715132 28627962

[B136] Popescu V. Klaver R. Voorn P. Galis-De Graaf Y. Knol D. L. Twisk J. W. R. (2015). What drives MRI-measured cortical atrophy in multiple sclerosis? *Mult. Scler.* 21 1280–1290. 10.1177/1352458514562440 25583833

[B137] Reich D. S. Lucchinetti C. F. Calabresi P. A. (2018). Multiple sclerosis. *N. Engl. J. Med.* 378 169–180. 10.1056/NEJMra1401483 29320652 PMC6942519

[B138] Reyes S. Smets I. Holden D. Carrillo-Loza K. Christmas T. Bianchi L. (2020). CSF neurofilament light chain testing as an aid to determine treatment strategies in MS. *Neurol. Neuroimmunol. Neuroinflamm.* 7:880. 10.1212/NXI.0000000000000880 32826298 PMC7455313

[B139] Rocca M. A. Parisi L. Pagani E. Copetti M. Rodegher M. Colombo B. (2014). Regional but not global brain damage contributes to fatigue in multiple sclerosis. *Radiology* 273 511–520. 10.1148/radiol.14140417 24927473

[B140] Romme Christensen J. Börnsen L. Khademi M. Olsson T. Jensen P. E. Sørensen P. S. (2013). CSF inflammation and axonal damage are increased and correlate in progressive multiple sclerosis. *Mult. Scler. J.* 19 877–884. 10.1177/1352458512466929 23178691

[B141] Romme Christensen J. Komori M. von Essen M. R. Ratzer R. Börnsen L. Bielekova B. (2019). CSF inflammatory biomarkers responsive to treatment in progressive multiple sclerosis capture residual inflammation associated with axonal damage. *Mult. Scler. J.* 25 937–946. 10.1177/1352458518774880 29775134 PMC6212343

[B142] Rosengren L. E. Karlsson J. E. Karlsson J. O. Persson L. I. Wikkelsø C. (1996). Patients with amyotrophic lateral sclerosis and other neurodegenerative diseases have increased levels of neurofilament protein in CSF. *J. Neurochem.* 67 2013–2018. 10.1046/j.1471-4159.1996.67052013.x 8863508

[B143] Sandelius Å Zetterberg H. Blennow K. Adiutori R. Malaspina A. Laura M. (2018). Plasma neurofilament light chain concentration in the inherited peripheral neuropathies. *Neurology* 90 e518–e524. 10.1212/WNL.0000000000004932 29321234 PMC5818017

[B144] Schneider R. Bellenberg B. Gisevius B. Hirschberg S. Sankowski R. Prinz M. (2021). Chitinase 3-like 1 and neurofilament light chain in CSF and CNS atrophy in MS. *Neurol. Neuroimmunol. Neuroinflamm.* 8:e906. 10.1212/NXI.0000000000000906 33172960 PMC7713721

[B145] Sejbaek T. Nielsen H. H. Penner N. Plavina T. Mendoza J. P. Martin N. A. (2019). Dimethyl fumarate decreases neurofilament light chain in CSF and blood of treatment naïve relapsing MS patients. *J. Neurol. Neurosurg. Psychiatry* 90 1324–1330. 10.1136/jnnp-2019-321321 31611264 PMC6902070

[B146] Sharma A. Petrillo M. Zhao G. Gagnon L. Plavina T. Singh C. (2018). “Strategic platform selection and validation of biomarker assays to measure serum neurofilament light and heavy chain in multiple sclerosis,” in *ECTRIMS Online Library* (Berlin).

[B147] Shimizu Y. Ota K. Ikeguchi R. Kubo S. Kabasawa C. Uchiyama S. (2013). Plasma osteopontin levels are associated with disease activity in the patients with multiple sclerosis and neuromyelitis optica. *J. Neuroimmunol.* 263 148–151. 10.1016/j.jneuroim.2013.07.005 23910387

[B148] Siffrin V. Vogt J. Radbruch H. Nitsch R. Zipp F. (2010). Multiple sclerosis - candidate mechanisms underlying CNS atrophy. *Trends Neurosci.* 33 202–210. 10.1016/j.tins.2010.01.002 20153532

[B149] Silber E. Semra Y. K. Gregson N. A. Sharief M. K. (2002). Patients with progressive multiple sclerosis have elevated antibodies to neurofilament subunit. *Neurology* 58 1372–1381. 10.1212/WNL.58.9.1372 12011283

[B150] Siller N. Kuhle J. Muthuraman M. Barro C. Uphaus T. Groppa S. (2019). Serum neurofilament light chain is a biomarker of acute and chronic neuronal damage in early multiple sclerosis. *Mult. Scler. J.* 25 678–686. 10.1177/1352458518765666 29542376

[B151] Sormani M. P. Haering D. A. Kropshofer H. Leppert D. Kundu U. Barro C. (2019). Blood neurofilament light as a potential endpoint in phase 2 studies in MS. *Ann. Clin. Transl. Neurol.* 6 1081–1089. 10.1002/acn3.795 31211172 PMC6562031

[B152] Tallantyre E. C. Bø L. Al-Rawashdeh O. Owens T. Polman C. H. Lowe J. (2009). Greater loss of axons in primary progressive multiple sclerosis plaques compared to secondary progressive disease. *Brain* 132 1190–1199. 10.1093/brain/awp106 19420101

[B153] Tartaglia M. C. Narayanan S. Francis S. J. Santos A. C. De Stefano N. Lapierre Y. (2004). The relationship between diffuse axonal damage and fatigue in multiple sclerosis. *Arch. Neurol.* 61 201–207. 10.1001/archneur.61.2.201 14967766

[B154] Thebault S. Abdoli M. Fereshtehnejad S. M. Tessier D. Tabard-Cossa V. Freedman M. S. (2020). Serum neurofilament light chain predicts long term clinical outcomes in multiple sclerosis. *Sci. Rep.* 10:10381. 10.1038/s41598-020-67504-6 32587320 PMC7316736

[B155] Thompson A. J. Banwell B. L. Barkhof F. Carroll W. M. Coetzee T. Comi G. (2018). Diagnosis of multiple sclerosis: 2017 revisions of the McDonald criteria. *Lancet Neurol.* 17 162–173. 10.1016/S1474-4422(17)30470-2 29275977

[B156] Tintore M. Rovira À Río J. Otero-Romero S. Arrambide G. Tur C. (2015). Defining high, medium and low impact prognostic factors for developing multiple sclerosis. *Brain* 138 1863–1874. 10.1093/brain/awv105 25902415

[B157] Tortorella C. Direnzo V. Taurisano P. Romano R. Ruggieri M. Zoccolella S. (2015). Cerebrospinal fluid neurofilament tracks fMRI correlates of attention at the first attack of multiple sclerosis. *Mult. Scler.* 21 396–401. 10.1177/1352458514546789 25168208

[B158] Trapp B. D. Peterson J. Ransohoff R. M. Rudick R. Mörk S. Bö L. (1998). Axonal transection in the lesions of multiple sclerosis. *N. Engl. J. Med.* 338 278–285. 10.1056/NEJM199801293380502 9445407

[B159] Tsuruha J. I. Masuko-Hongo K. Kato T. Sakata M. Nakamura H. Sekine T. (2002). Autoimmunity against YKL-39, a human cartilage derived protein, in patients with osteoarthritis. *J. Rheumatol.* 29 1459–1466. 12136906

[B160] van der Vuurst de Vries R. M. Wong Y. Y. M. Mescheriakova J. Y. van Pelt E. D. Runia T. F. Jafari N. (2019). High neurofilament levels are associated with clinically definite multiple sclerosis in children and adults with clinically isolated syndrome. *Mult. Scler. J.* 25 958–967. 10.1177/1352458518775303 29774770 PMC6545618

[B161] Varhaug K. N. Torkildsen Ø Myhr K. M. Vedeler C. A. (2019). Neurofilament light chain as a biomarker in multiple sclerosis. *Front. Neurol.* 10:338. 10.3389/fneur.2019.00338 31024432 PMC6460359

[B162] Vogt M. H. J. Lopatinskaya L. Smits M. Polman C. H. Nagelkerken L. (2003). Elevated osteopontin levels in active relapsing-remitting multiple sclerosis. *Ann. Neurol.* 53, 819–822. 10.1002/ana.10606 12783433

[B163] Watanabe M. Nakamura Y. Michalak Z. Isobe N. Barro C. Leppert D. (2019). Serum GFAP and neurofilament light as biomarkers of disease activity and disability in NMOSD. *Neurology* 93 E1299–E1311. 10.1212/WNL.0000000000008160 31471502

[B164] Williams T. Zetterberg H. Chataway J. (2020). Neurofilaments in progressive multiple sclerosis: a systematic review. *J. Neurol.* 1:3. 10.1007/s00415-020-09917-x 32447549 PMC8357650

[B165] Xu Z. Henderson R. D. David M. McCombe P. A. (2016). Neurofilaments as biomarkers for amyotrophic lateral sclerosis: a systematic review and meta-analysis. *PLoS One* 11:e0164625. 10.1371/journal.pone.0164625 27732645 PMC5061412

[B166] Yin X. Crawford T. O. Griffin J. W. Tu P. H. Lee V. M. Y. Li C. (1998). Myelin-associated glycoprotein is a myelin signal that modulates the caliber of myelinated axons. *J. Neurosci.* 18 1953–1962. 10.1523/jneurosci.18-06-01953.1998 9482781 PMC6792914

[B167] Yuan A. Rao M. V. Veeranna Nixon R. A. (2012). Neurofilaments at a glance. *J. Cell Sci.* 125 3257–3263. 10.1242/jcs.104729 22956720 PMC3516374

[B168] Ziemssen T. Akgün K. Brück W. (2019). Molecular biomarkers in multiple sclerosis. *J. Neuroinflamm.* 16:272. 10.1186/s12974-019-1674-2 31870389 PMC6929340

[B169] Zucchi E. Bonetto V. Sorarù G. Martinelli I. Parchi P. Liguori R. (2020). Neurofilaments in motor neuron disorders: towards promising diagnostic and prognostic biomarkers. *Mol. Neurodegener.* 15:58. 10.1186/s13024-020-00406-3 33059698 PMC7559190

